# *In Vivo* Dynamic Deformation of Articular Cartilage in Intact Joints Loaded by Controlled Muscular Contractions

**DOI:** 10.1371/journal.pone.0147547

**Published:** 2016-01-25

**Authors:** Ziad Abusara, Markus Von Kossel, Walter Herzog

**Affiliations:** Human Performance Laboratory, Faculty of Kinesiology, University of Calgary, Alberta, Canada; University of Rochester, UNITED STATES

## Abstract

When synovial joints are loaded, the articular cartilage and the cells residing in it deform. Cartilage deformation has been related to structural tissue damage, and cell deformation has been associated with cell signalling and corresponding anabolic and catabolic responses. Despite the acknowledged importance of cartilage and cell deformation, there are no dynamic data on these measures from joints of live animals using muscular load application. Research in this area has typically been done using confined and unconfined loading configurations and indentation testing. These loading conditions can be well controlled and allow for accurate measurements of cartilage and cell deformations, but they have little to do with the contact mechanics occurring in a joint where non-congruent cartilage surfaces with different material and functional properties are pressed against each other by muscular forces. The aim of this study was to measure *in vivo*, real time articular cartilage deformations for precisely controlled static and dynamic muscular loading conditions in the knees of mice. Fifty and 80% of the maximal knee extensor muscular force (equivalent to approximately 0.4N and 0.6N) produced average peak articular cartilage strains of 10.5±1.0% and 18.3±1.3% (Mean ± SD), respectively, during 8s contractions. A sequence of 15 repeat, isometric muscular contractions (0.5s on, 3.5s off) of 50% and 80% of maximal muscular force produced cartilage strains of 3.0±1.1% and 9.6±1.5% (Mean ± SD) on the femoral condyles of the mouse knee. Cartilage thickness recovery following mechanical compression was highly viscoelastic and took almost 50s following force removal in the static tests.

## Introduction

Articular cartilage is a hydrated fibre composite material that covers the articular surfaces of bones in synovial joints. It consists of cells (chondrocytes) that occupy 2–15% of the volumetric fraction, and an intercellular matrix (85–98% of total volumetric fraction) with 65–80% water content [1;2]. The primary functions of articular cartilage include the transmission and distribution of forces to minimize stress concentrations, and to provide smooth areas for the gliding of articulating joint surfaces. Mechanical loading of joints, and the associated cartilage deformations, have been implicated as primary reasons for the development and progression of osteoarthritis (OA) [3]. The deformational behavior of articular cartilage from excised tissue samples has been investigated thoroughly in confined and unconfined compression with metallic indentation devices [4–6]. In addition, cartilage deformations have been calculated based on theoretical tissue models [7–13] in an attempt to understand joint biomechanics. *In-vivo* studies have been performed using MR imaging to describe changes in thickness of knee joint cartilage after activities such as bending, normal gait, and squatting [14–16]. These previous studies were limited to measuring cartilage deformations during steady-state conditions following a loading protocol and although they likely reflect the cartilage response to physiological loading conditions, they are not time-sensitive enough to measure the continuous cartilage deformations during the mechanical loading of a joint.

Cartilage deformation is known to cause deformations of the chondrocytes and their nuclei [4;17–20], and these deformations, in turn, are known to affect the biological signaling response of chondrocytes that control the maintenance and adaptation of the tissue [21–25]. However, the pathways from joint loading, to global and local cartilage deformation, the associated cell deformations, and the corresponding cellular responses remain unexplored in intact joints, in part due to the difficulties of loading joints in a controlled, physiological manner and simultaneously measuring cell responses.

Recently, we developed a novel *in vivo* testing system that allows for controlled loading of mouse knees through muscular contraction and permits the quantification of the associated chondrocyte deformations. This system has also been used for analyzing changes in synovial fluid composition following controlled loading of knees [18;26]. It can also be used to measure chondrocyte signaling responses associated with joint loading. Results from these studies showed that chondrocyte mechanics are different in joints compared to the traditional *in situ* and *in vitro* approaches. For example, cells deform quickly (within seconds) upon joint loading but take minutes to recover their original, pre-load shapes following load removal [18]. In contrast, chondrocytes removed from the cartilage and seeded in gel constructs recover virtually instantaneously following load removal, thereby exhibiting nearly elastic behaviour [27]. Furthermore, changes in synovial fluid composition associated with joint loading can be measured and long term cartilage adaptations or degenerations can be observed in the context of cartilage and cell mechanics [26]. However, except for pilot results, little is known about the mechanics of articular cartilage inside a joint loaded by physiologically relevant and controlled muscular contractions, and although some work on cartilage deformations in loaded human knees have been performed [28–31], these studies are restricted to static and near steady-state conditions because of the limited time resolution of magnetic resonance imaging. The biomechanics of dynamic cartilage behavior and properties in intact joints remain unexplored.

The aim of this study was to measure *in vivo*, real time articular cartilage deformations for precisely controlled static and dynamic muscular loading conditions in joints. We chose the mouse knee as our experimental model.

## Materials and Methods

### Animal preparation

**Fig 1:**
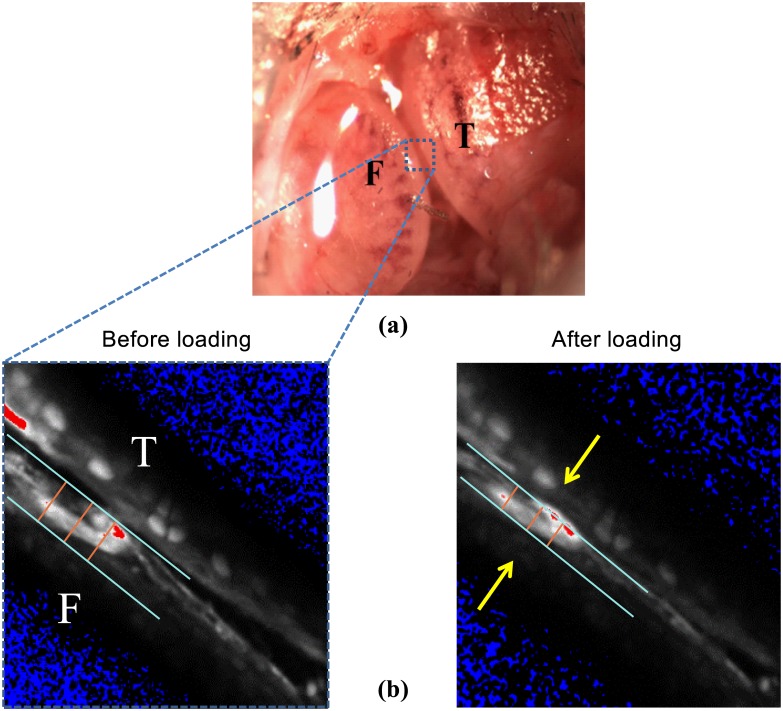
(a) Exposed mouse knee preparation showing the medial tibial plateau (T), and the medial femoral condyle (F) with the meniscus removed. (b) Tibio-femorol cartilage-on-cartilage space decreased to zero during muscular loading. Arrows show cartilage deformation area.

This study was carried out in accordance with the guidelines of the Canadian Council on Animal Care and was approved by the committee for Animal Use Ethics at the University of Calgary. Thirteen C57 adult male mice (10–12 weeks of age, 28 ±2g body weight) were used in this study. Mice were anesthetized with an isoflurane/oxygen mixture (1–3%). The left knee joint was shaved and secured in a stereo-taxic frame that was rigidly attached to the stage of a dissecting microscope. The medial aspect of the joint was exposed with a 6 mm incision just posterior to the medial collateral ligament. The articular capsule was carefully released and the medial meniscus was excised to provide a direct view of the medial tibio-femoral joint. The exposed medial aspect of the knee was then washed and filled with 30μl of prepared Vybrant CFDA-SE (excitation 490nm, emission 520nm, Molecular Probes/ Invitrogen, USA). After a 30 minute incubation period in complete darkness, excess stain was removed and the area was washed and filled with a fresh phosphate buffered saline solution (PBS) allowing for the use of a water-immersion objective (Fig 1a).

### Two-photon microscopy

The anesthetized mouse was then moved onto the stage of a dual photon microscope (LSM 510, Zeiss Inc., Germany). Medial femoral condyle cartilage was imaged using a 40×1.00 NA water-immersion objective (Zeiss Inc., Germany) coupled with a Chameleon XR infrared laser (Coherent Inc., USA). Two photon microscopy was used so that we could penetrate the entire medial compartment cartilage and to reduce photo bleaching and photo damage encountered with regular confocal microscopy [32].

### Muscular loading of the mouse knee

Controlled muscular loading of the knee was achieved by stimulation of the knee extensor muscles using two fine wire electrodes inserted into the quadriceps group. Muscles were stimulated using electrical stimulation with a Grass (S8800) digital stimulator [33]. The free tips of the exposed fine wires were separated by 2 mm, and application of 2–7 volts resulted in forces ranging from 15–80% of maximal [26]. Maximal stimulation was typically reached at <9 volts. Knee extensor torques were measured with a strain bar (Entran Sensors & Electronics, USA) attached to the distal part of the tibia while the femur was rigidly fixed to avoid any movement (<0.5 μm) [18].

#### Single static load

**Fig 2:**
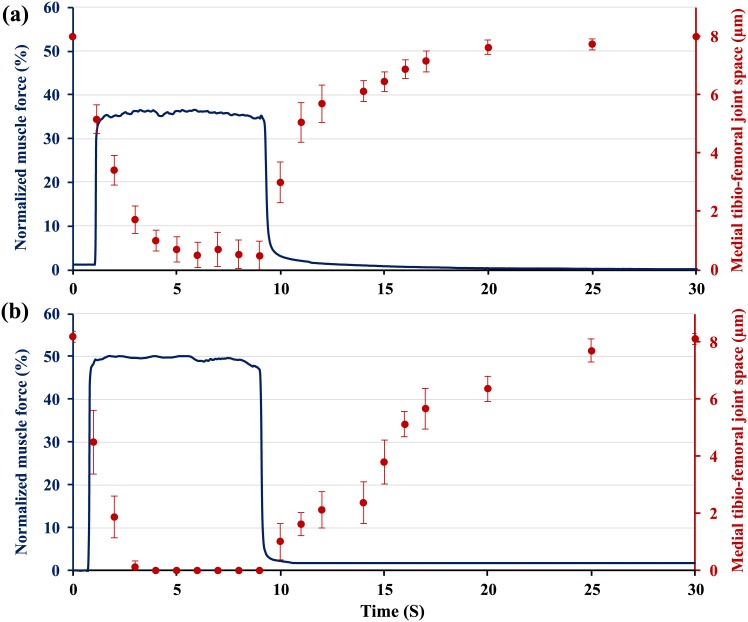
Normalized (relative to maximum = 100%) knee extensor forces as a function of time. The medial tibio-femoral joint space narrowed during loading with (a) 35% of muscular loading (n = 7), the two cartilage surfaces never touch and no sign of cartilage deformation was noticed at this force. (b) 50% of muscular loading (n = 7), the cartilage-on-cartilage space reached zero and cartilage thickness decreased for these loading conditions. In both cases, medial tibio-femoral cartilage-on-cartilage space returned back to its original position in ~20s following the removal of force.

**Fig 3:**
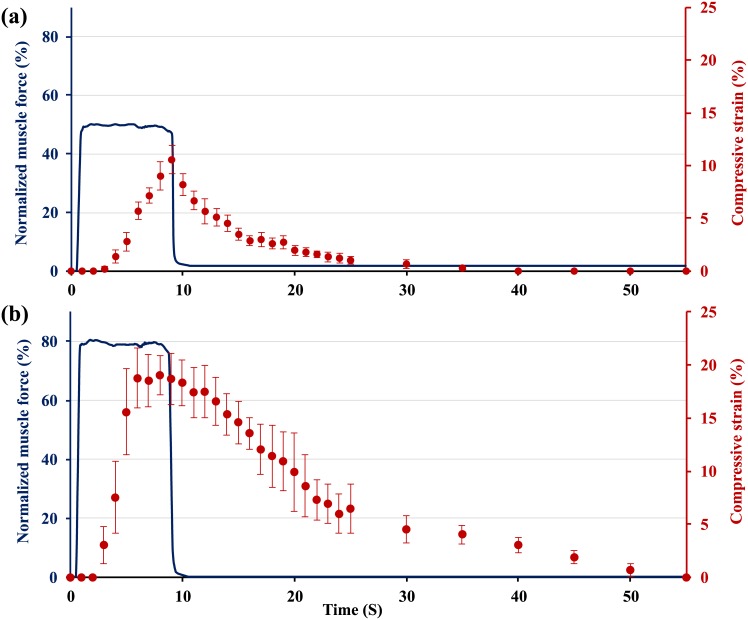
Normalized (relative to maximum = 100%) knee extensor forces as a function of time. Muscles were stimulated for 8s at a voltage and frequency producing approximately (a) 50% of the maximal muscle force, cartilage compressive strain (mean ±1SD; n = 7) increased almost linearly 3s after the force application. Cartilage takes 20s for full recovery upon unloading. (b) 80% of the maximal isometric force. Cartilage compressive strain (mean ±1SD; n = 7) increased rapidly and reached near steady state conditions at 6s following force application. Cartilage tissue recovered to its original shape within approximately 45s following force removal.

A single static compressive load equivalent to 40, 50, 60, 70 and 80% of the maximal knee extensor muscle force was applied to the knee for 8s (n = 7; Figs 2 and 3). Two-photon image sections were recorded every second before loading, during the 8s loading period, and for the first 10s following the removal of the load. After the first ten seconds of recovery, images were recorded every 5s for up to 60s following load removal (Figs 2 and 3). The medial tibio-femoral joint space was measured during muscular loading until the two cartilage surfaces started to touch (Fig 2). Then, femoral cartilage deformations were measured (Fig 3).

#### Dynamic cyclic loading

**Fig 4:**
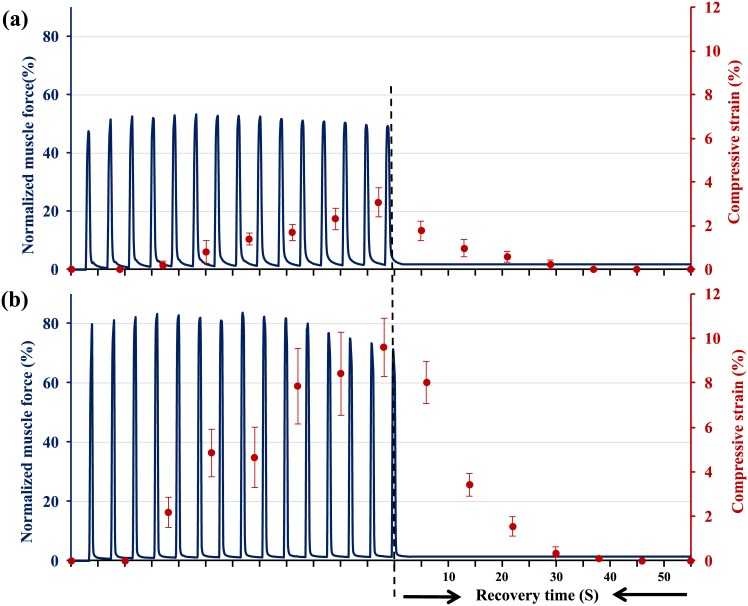
Normalized (relative to maximum = 100%) knee extensor forces as a function of time. Muscles were stimulated 15 times for 0.5s every 4s at a voltage and frequency producing approximately (a) 50% of the maximal muscle force. Cartilage compressive strain (mean ±1SD; n = 6) increased as a function of the number of muscular contractions. Full cartilage recovery takes around 20s upon unloading. (b) Loading using approximately 80% of the maximal isometric force. Cartilage strain (mean ±1SD; n = 6) increased with increasing the number of muscular contractions and reached its maximum at the last contraction. Cartilage tissue recovered to its original shape within approximately 35s following force removal.

Stimulation strain of 0.5s at 50 Hz every 4s for 15 repeat contractions were applied to the articular surfaces (n = 6) using compressive loads of 40, 50, 60, 70 and 80% of the maximal knee extensor force. Two- photon scans were taken before loading, in the rest period (unloaded) following every second contraction, and every 10s following cessation of muscular stimulation (Fig 4). Total compressive tissue strains were quantified as described below.

### Two-photon image analysis

Prior to starting the muscular loading protocol, a stack of images at intervals of 1μm was acquired for the medial compartment of the knee moving from medial to lateral across the joint. The minimum distance between the cartilage surfaces of the opposing femoral condyle and tibial plateau was measured for each 2D slice using commercial software (Image-Pro V6.3, Media Cybernetics, Inc., USA). The 2D slice containing the smallest tibio-femoral cartilage-to-cartilage distance across the medial compartment of the joint was identified (typically about 8 μm), the stage position was locked at that location, and all measurements during the mechanical loading of the knee were made at this position.

#### Cartilage deformation

Cartilage deformations for the static and dynamic loading conditions were measured by acquiring a time series of images (pixel size: 0.41 μm×0.41 μm; pixel dwell time: 1.61 μs; frame scan time: 0.988 s). Cartilage thickness measurements were made at a single location using a 2D slice through the medial tibio-femoral joint. The 2D slice was always located in the medial tibio-femoral joint at the location where the distance between opposing cartilage surfaces was a minimum in the unloaded condition. Using the 2D slice image, the medial tibio-femoral joint space and cartilage deformation were measured using Image-pro software. On each image, two lines indicating the femoral cartilage top (surface) and bottom (tide line) were drawn and cartilage thickness was defined as the perpendicular distance between these two parallel lines (Fig 1b).

Three points were taken on each of the two lines indicating the top and bottom of the femoral condyle cartilage: one in the middle of the deformation (the point of greatest deformation), and two points at 10μm on either side of this mid-point (Fig 1b). Cartilage deformation was defined as the average deformation at these three points from the resting position. In order to assess the inter-examiner repeatability of our cartilage thickness measurements, two independent examiners carried out all analyses in duplicate. When the two measurements differed by more than 0.5μm (which happened in less than 5% of the cases), the measurement was carefully repeated by both examiners until agreement (within 0.5μm) was reached. Compressive strains and tissue strains were calculated as engineering strains (ε = (h-h_0_)/h_0_, where h is the deformed tissue thickness and h_0_ is the tissue thickness prior to loading).

### Statistical analysis

Statistical analysis was performed using the non-parametric Mann-Whitney test for static and dynamic load at each of the determined target forces. Statistical significance was set *a priori* at α = 0.05. Results are presented as means and ± 1 standard deviation (SD).

## Results

### Single static load

The average overall cartilage thickness was 32±2 μm on the medial femoral condyles. For forces equivalent to about 35% of the maximal isometric knee extensor strength (Fig 2a), the medial tibio-femoral cartilage-on-cartilage space did not close completely. Contact between opposing cartilage surfaces was made at forces of approximately 40% of the maximal muscular force with no measurable cartilage deformation. With increasing forces, the cartilages started to deform. For example, for a force equivalent to about 50% of maximal, the opposing surfaces touched after about 3s, followed by cartilage deformation (Fig 2b).

Fifty and 80% of maximal muscular forces (equivalent to approximately 0.4N and 0.6N respectively) produced average peak articular cartilage compressive strains for an 8s contraction of 10.5±1% and 18.3±1.3% (Mean ± SD) respectively (Fig 3). Following cartilage contact, cartilage compressive strains increased and reached peak values at the end of force application (Fig 3). Cartilage tissue recovered to its original thickness within approximately 25s for 50% force, and 50s for the 80% force (Fig 3).

### Dynamic cyclic load

Articular cartilage compressive strains increased as a function of muscular load (Fig 4). Fifty and 80% of the maximal muscular forces produced average peak articular cartilage strains of 3.0±1.1% to 9.6±1.5% (Mean ± SD), respectively (Fig 4). Cartilage tissue recovered to its original shape within approximately 20 and 30s following force removal for the 50 and 80% of the total maximal forces, respectively (Fig 4).

**Fig 5:**
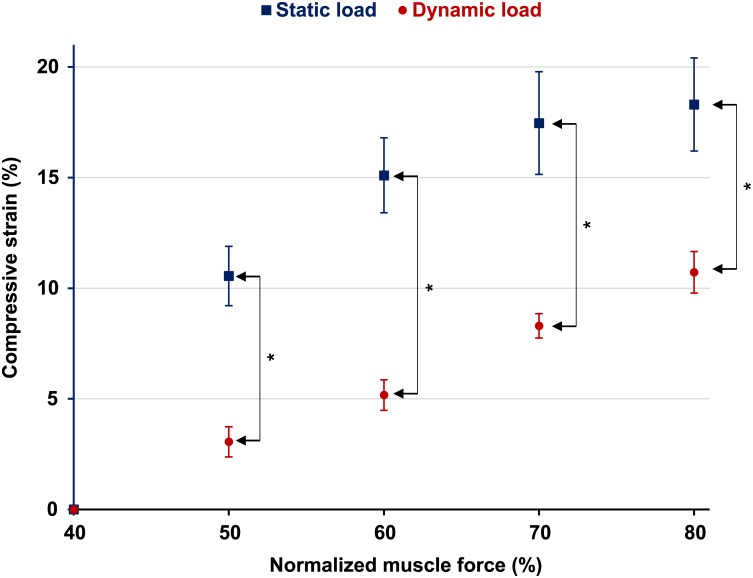
Peak compressive strains as a function of normalised muscle force for static and dynamic loading. For static loading, articular cartilage surfaces do not touch below approximately 40% of maximal muscle force, then cartilage strain increased between about 40–70% of the maximal force, and remained almost constant from 70–80% of maximal force (n = 7). For dynamic loading, articular surfaces did not touch below ~40% of the maximal isometric muscle force. Beyond ~40% force porduction, cartilage strains increased almost lineary between 50–80% of the maximal force (n = 6). Vertical bars on the side show significant differences between groups.

Increases in muscular loading of the knee caused an increase in articular cartilage deformation (Fig 5). The 80% of maximal force contractions were the highest forces that could be maintained for the static and dynamic loading conditions without obvious fatigue and associated decline of force during testing. Therefore only results between 50 and 80% of the total maximal muscular forces are shown here (Fig 5).

## Discussion

In this study, we investigated the real-time deformations of articular cartilage in murine knee joints using an *in vivo* testing system coupled with two-photon laser microscopy. The results of this study suggest that tibio-femoral contact between the articulating surfaces is only achieved with relatively high muscular forces (approximately 40% of maximal or 1.3 x body mass of the mouse). This result may possibly be an artifact of the current setup where the medial meniscus was removed, and physical contact between the two articulating surfaces was not present at zero muscular force. Furthermore, the lack of cartilage-on-cartilage contact may also be caused by an increase in joint laxity due to the partial release of the MCL, or valgus loading caused by the loss of medial soft tissue structures. In the fully intact joint, contact may occur at lower forces than observed in our preparation. This finding will need to be investigated further. However, our result suggests, that in the presence of medial meniscectomy, high forces are required to obtain physical cartilage to cartilage contact in the medial tibio-femoral compartment of the mouse knee.

The results of the current study may possibly shed light on findings from previous work on protein concentration changes in synovial fluid with joint loading [26]. In that previous study, we found that the total protein concentration in synovial fluid of the mouse knee did not change when muscle forces were kept below approximately 50% of the maximal isometric knee extensor force. On the other hand, muscle forces greater than about 55% of the maximal isometric force produced a consistent and significant increase in total synovial fluid protein concentration, including a significant increase in PRG4 [26]. Taken together, these results suggest that changes in synovial fluid composition may be triggered by cartilage and associated chondrocyte deformations. If muscular forces are below those causing cartilage/cell deformation (about 50% of maximal knee extensor force), then the synovial fluid remains unchanged. However, when cartilage/cell deformation occurs, chondrocytes may release proteins (including PRG4) thus changing the composition of the synovial fluid. This result is further supported by previous experiments in which blocking of chondrocyte release channels prevented changes in synovial fluid composition even at high muscular loads [26].

Cartilage behaves highly visco-elastically in joints. Specifically, peak cartilage deformation is reached much later than peak joint loads. Here, we found that peak cartilage deformations in the dynamic loading experiments were reached at the end of the 15 cyclic contractions, suggesting that cartilage deformation would have been greater than found here had joint loading been applied for longer than 15 contractions. This result is in agreement with other reports [34] using cartilage explants where it was found that the number of loading cycles required to achieve a steady-state deformation response in normal bovine articular cartilage was at least 33 cycles.

The visco-elastic cartilage deformation observed here suggests that in a normal cyclic movement, such as walking (loading time of approximately 800ms during each step), peak steady-state cartilage deformations are only reached after multiple step cycles. Similarly, full cartilage thickness recovery following the static and dynamic loading conditions using 50% of maximal isometric knee extensor force took approximately 20 to 25s (Figs 3 and 4), suggesting that deformation recovery within cyclic movements, such as walking, with an unloading time of less than 1s, may be neglected. Our results also agree with the work by Eckstein et al [35] who showed that it took 50 knee bends to get maximal cartilage deformations, and it took around 90 minutes for the cartilage to recover its thickness loss.

Chondrocyte deformation has been linked closely to cartilage deformation [36]. The results of this study are consistent with earlier findings on chondrocyte mechanics in the knees of live mice [18] and the intact cartilage attached to its native bone [37]. In the first study, we showed that chondrocytes deform within seconds but take several minutes for full recovery of their original, pre-load shape. In the second study, the authors indicated that full recovery of compressed chondrocytes takes more than five minutes. In both of these cases, chondrocytes showed a highly viscoelastic behavior following load removal. The slow recovery of cells following load removal has the advantage that cells do not undergo big deformations for each loading cycle. Rather, cells seem to deform greatly for the first few loading cycles and then reach a near steady-state shape for subsequent loading cycles. This finding naturally leads to the speculation that the magnitude of cell signalling is greatest for the first few loading cycles, only to fade to zero in subsequent loading cycles. However, how cell deformations and cell signaling are related can only be fully understood by measuring the mechanics and signaling of chondrocytes simultaneously, which has not been done to date in cells fully embedded in their native environment. Such combined mechanical and signaling work also needs to be done in osteoarthritic joints in order to gain an understanding of how mechanical loads affect cartilage adaptation and joint degeneration leading to mechanically induced osteoarthritis.

Cartilage deformation in the present setup seems to reach a plateau-like state at 6s following loading that is equivalent to 80% of the maximal knee extensor muscular force (Fig 3b). This indicates that at high *in vivo* muscular forces only few seconds are necessary for cartilage to reach a plateau-like deformation state, and that additional loading does not cause much further deformation of the tissue. Our result agrees with *in vivo*, real time cartilage compressive strain measurements made in the human ankle [38] and human tibiofemoral joints [39]. The authors of the first study found that peak compressive strains (27±6%) were reached 15s after the onset of loading. In the second study, peak cartilage deformations increased rapidly within the first 20s of loading (10.5±0.8%) and remained relatively constant thereafter. Both studies used forces of approximately 1x body weight for joint compression. The 80% muscular force used in the present study was about 0.6N, or approximately 2x body weight of the mouse, and thus is relatively higher than that used by Li et al. [38] for the human ankle and Hosseini et al. [39] for the human tibiofemoral joint. This difference in load amplitude might explain why the mice femoral cartilage reached a plateau-like deformation state faster (6s) than the time for reaching steady-state conditions in the human studies. Other factors, such as differences in cartilage matrix composition and permeability, might also explain the small differences in time to steady-state cartilage deformation between our study and those in human ankles and knees.

One of the limitations of this study was that the cartilage thickness measurements were made at a location using a 2d slice through the medial tibio-femoral joint. Therefore, the values reported here are applicable only for that particular slice location, and so the maximum deformation of the articular cartilage might be under estimated.

## Conclusion

We developed a novel in vivo system that allows for continuous measurement of articular cartilage deformations following static and dynamic muscular loading in the knee of live mice. Articular cartilage deforms substantially for loading conditions that are well within the physiological range. Furthermore, cartilage behaves highly visco-elastically in vivo, suggesting that deformation recovery within cyclic movements such as walking, with an unloading time of less than 1s, may probably be safely neglected. Our results may serve as baseline data for direct validation of finite element models of cartilage deformation for well characterized loading conditions. Our findings may also be used for comparing tissue (cartilage) mechanical responses with the corresponding cell mechanical responses for physiologically relevant loading conditions.
